# Ribosomal DNA gene copies are increased in blood and brain of Japanese schizophrenia patients

**DOI:** 10.1371/journal.pone.0280694

**Published:** 2023-01-20

**Authors:** Sen Li, Ikuo Otsuka, Takaki Tanifuji, Satoshi Okazaki, Tadasu Horai, Motonori Takahashi, Takeshi Kondo, Yasuhiro Ueno, Akitoyo Hishimoto

**Affiliations:** 1 Department of Psychiatry, Kobe University Graduate School of Medicine, Kobe, Japan; 2 Division of Legal Medicine, Department of Community Medicine and Social Health Science, Kobe University Graduate School of Medicine, Kobe, Japan; 3 Department of Psychiatry, Yokohama City University Graduate School of Medicine, Yokohama, Japan; McGill University Department of Psychiatry, CANADA

## Abstract

Past evidence has indicated increased ribosomal DNA (rDNA) content in the blood of patients with schizophrenia (SCZ) among European populations. Here, for the first time, we investigated the rDNA copy number (rDNAcn) of SCZ in East Asian populations as well as in blood and brain tissues. In this study, we measured 18S/28S rDNAcn in the peripheral blood of live participants (81 patients with SCZ and 98 healthy controls) and the dorsolateral prefrontal cortices (DLPFCs) of postmortem individuals (10 patients with SCZ and 23 non-psychiatric controls) in the Japanese population. Patients with SCZ had significantly increased 18S/28S rDNAcn in the blood compared to controls (p < 0.05). 18S rDNAcn was significantly increased in the brain of patients with SCZ compared to controls (p < 0.05). In conclusion, regarding the increased rDNAcn in the blood of patients with SCZ that was previously reported in Europeans, we successfully replicated this by using a different, ethnically East Asian, cohort. Additionally, we provide the first evidence of increased rDNAcn in the brain of patients with SCZ. These findings may help to elucidate the molecular underpinnings of SCZ pathophysiology related to ribosomal DNA abnormalities.

## Introduction

Ribosomes are critical structures that exist in all living organisms and act as protein synthesis sites. The ribosome is composed of a core complex of four ribosomal ribonucleic acid (RNA) molecules (18S, 5.8S, 28S, and 5S) and approximately 70–80 proteins [1]. In humans, multi-copy genes encoding ribosomal RNA are located on five acrocentric autosomal chromosomes (chr13, 14, 15, 21, and 22), forming ribosomal deoxyribonucleic acid (rDNA). Indeed, rDNA dynamics, such as rDNA copy number (rDNAcn) are of particular interest because they can affect the rate of ribosomal RNA biosynthesis, which is the main limiting factor of ribosome biogenesis [2].

Several lines of evidence have indicated that rDNA transcriptional activity may play a key role in neuronal plasticity. Dysfunctional neural plasticity has been proposed as a key pathophysiological mechanism in schizophrenia (SCZ), which is associated with genetic factors [3]. Some studies have shown changes in the transcriptional activity of rDNA in the brains and lymphocytes of patients with SCZ [4–6]. Given this evidence, abnormalities in rDNAcn may contribute to the development of SCZ. However, there are just two studies reported by one research group regarding the association between rDNAcn and SCZ in European patients (these documented increased rDNAcn in the SCZ lymphocytes) [7, 8], while no study focused rDNAcn in brain tissues of patients with SCZ.

In this study, using the Japanese SCZ cohort, we tested whether the rDNAcn of peripheral blood from East Asian patients with SCZ was increased, similar to European patients with SCZ. Furthermore, because most psychiatric problems, including SCZ, may be derived from the main diathesis in specific brain regions, we ascertained whether the aberrant rDNA content also occurs in postmortem brain tissues of patients with SCZ.

## Materials and methods

### Ethics statements

The study design and related procedures were performed in accordance with the Declaration of Helsinki. This study was approved by the Ethical Committee for Genetic Studies of Kobe University Graduate School of Medicine (Approval No. 170203). Written informed consent was obtained from all living participants and the families of deceased individuals from whom post-mortem brain samples were obtained.

### Subjects

Whole blood samples were obtained from living patients with SCZ (n = 81) without severe physical illness and healthy controls (n = 98) through peripheral vein and stored in EDTA tube. Autopsied brains were obtained from 10 patients with SCZ and 23 non-psychiatric control individuals. All the participants were of Japanese descent. Patients with SCZ were diagnosed by a psychiatrist according to the Diagnostic and Statistical Manual of Mental Disorders-IV (DSM-IV) criteria based on unstructured interviews and reviews of their medical records. Among the 81 patients with SCZ whose blood samples were obtained, Global Assessment Functioning (GAF) scores [9] and Brief Psychiatric Rating Scale (BPRS) [10] were obtained from 60 patients (the details for GAF and BPRS are shown in **S1 Table in S1 File**), and the information on antipsychotic dose calculated with chlorpromazine equivalents at blood draw was obtained from 78 patients. A total of 98 healthy living controls were recruited from the main islands of Japan, including medical students, hospital workers, and individuals from the general population. None of the control participants were related to each other or manifested psychiatric problems in the unstructured interviews. During the interview, all control participants were checked for personal and family history of psychiatric disorders based on the DSM-IV, and those with a personal or family history of psychiatric disorders were excluded. Autopsies were conducted at the Division of Legal Medicine in the Department of Community Medicine and Social Health Science at Kobe University Graduate School of Medicine. The dorsolateral prefrontal cortex (DLPFC) was dissected on dry ice for DNA extraction. The demographic and clinical data of the cohort are shown in **Table 1**.

**Table 1:** Demographic and clinical details of the subjects.

	Peripheral blood from living subjects			DLPFC from postmortem subjects		
	Schizophrenia (n = 81)	Control (n = 98)	p^a^	Schizophrenia (n = 10)	Control (n = 23)	p^a^
Average age in years (± s.d.)	36.1 (6.8)	33.5 (8.7)	0.03	57.3 (19.2)	56.9 (16.6)	0.95
Sex (Male/Female)	38/43	40/58	0.45	5/5	15/8	0.46
PMI in hours (± s.d.)				20.2 (7.6)	18.1 (10.0)	0.58
Clinical information^b^						
GAF score (± s.d.)	37.0 (11.9)					
BPRS (± s.d.)	53.4 (15.3)					
Antipsychotic dose (± s.d.)	928.9 (710.7)					

Abbreviations: s.d., standard deviation; PMI, post-mortem interval; GAF, Global Assessment Functioning; BPRS, Brief Psychiatric Rating Scale.

^a^ p-values to estimate difference between cases and controls were calculated using Mann-Whitney tests or Chi-squared tests.

^b^ GAF scores and BPRS were obtained from 60 schizophrenia patients, and the information on antipsychotic dose were obtained from 78 patients.

### Assessment of rDNAcn in blood and brain samples

Blood and brain samples were stored at −80°C before use. DNA was extracted using the FlexiGene DNA Kit (Qiagen Inc., Valencia, CA, USA). The quantity and purity of extracted DNA was determined using a NanoDrop spectrophotometer (Thermo Scientific, Wilmington, DE, USA). Measurement of rDNAcn was done by quantitative polymerase chain reaction (qPCR) using standard curve base analysis by applying a previously reported method with minor modifications [11]. To estimate rDNAcn, amplicons targeting 18S and 28S coding regions of rDNA were used, with amplicons corresponding to the coding regions of the albumin gene (ALB) as an endogenous control. rDNAcn was determined as the 18S or 28S / ALB ratio by measuring the ratio of ribosomal DNA to that of a reference endogenous control gene in each sample. The primer sequences and amplification conditions are listed in **S2 Table in S1 File**. All qPCR experiments were performed with a 7500 Real-Time PCR System (Applied Biosystems, Foster City, CA), with SYBR Green Master Mix (Applied Biosystems, Foster City, CA). Each sample was run in triplicate using 10 ng of DNA. Laboratory personnel performing the assays were blinded to case-control status and demographic data. The sample order was randomized for each batch.

### Statistical analysis

Statistical analyses were performed using R version 3.6.3 (https://www.r-project.org). The Kolmogorov-Smirnov test was used to assess the normality of the data. Mann-Whitney U tests were performed to analyze between-group comparisons of continuous variables. Regression analyses using generalized linear models (gamma distribution and log link) or multiple linear regression were applied to analyze between-group comparisons of rDNAcn, with covariates (age and sex), as needed. Spearman’s rho tests were performed to assess the relationship between 18S rDNAcn and 28S rDNAcn. Dummy variables were used as necessary (phenotype, control = 0 and SCZ = 1; sex, male = 0 and female = 1). Statistical significance was defined as a two-tailed p < 0.05.

## Results

The individual values for the 18S and 28S rDNAcn of blood were strongly correlated in healthy controls (Spearman’s rho = 0.76, p < 0.001) and moderately correlated in patients with SCZ (Spearman’s rho = 0.57, p < 0.001) (**S1 Fig in S1 File**). The individual values for the 18S and 28S rDNAcn of DLPFCs were strongly correlated in the non-psychiatric controls (Spearman’s rho = 0.92, p < 0.001) and patients with SCZ (Spearman’s rho = 0.84, p = 0.002) (**S2 Fig in S1 File**).

**Fig 1:**
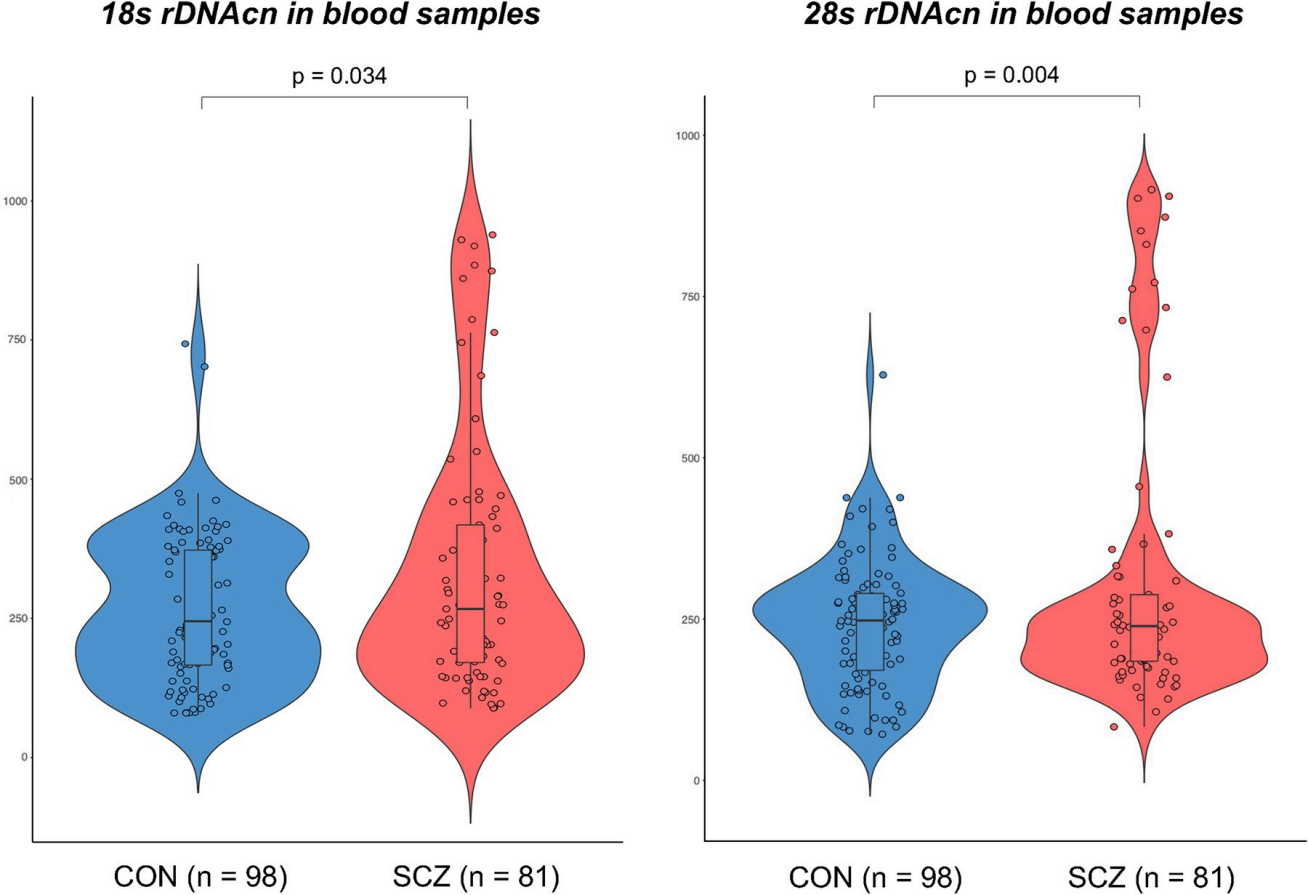
Violin and scatter dot plot including box and whiskers (with minimum to maximum) of 18S/28S ribosomal DNA copy number (rDNAcn) in blood samples from patients with schizophrenia (SCZ) and controls (CON). All p values were adjusted for age and sex as covariates.

The distribution of 18S/28S rDNAcns in the blood samples was skewed (Kolmogorov–Smirnov test, p < 0.05). The results of regression analyses with a generalized linear model of 18S/28S rDNAcn in blood samples of patients with SCZ (n = 81) and healthy controls (n = 98) with age and sex as covariates are shown in **S3 Table in S1 File**. These analyses showed that patients with SCZ had significantly increased rDNAcn in the blood compared to controls for 18S rDNAcn [SCZ: mean ± standard deviation (SD) = 331.6 ± 229.5, min/max = 85.3/946.2; CON: 264.3 ± 133.6, 76.4/748.1; p = 0.034] and 28S rDNAcn (SCZ: mean ± SD = 296.7 ± 219.4, min/max = 67.8/912.5; CON: 228.4 ± 100.0, 56.2/621.1; p = 0.004), respectively (**Fig 1**).

In the subgroup analysis, while high GAF scores in patients with SCZ had a nominal trend association with increased 18S rDNAcn (p = 0.096), low BPRS in patients with SCZ had a significant association with increased 18S rDNAcn (p = 0.020). In contrast, 28S rDNAcn in patients with SCZ did not have any association with GAF scores and BPRS. Antipsychotic doses did not show a significant association with 18S/28S rDNAcn in patients with SCZ (**Table 2**).

**Table 2:** Results of regression analysis between rDNAcn of peripheral blood and the clinical demographics in patients with SCZ.

	18s rDNAcn					28s rDNAcn			
	β^a^		s.e.	t	p^b^	β^a^	s.e.	t	p^b^
***Total samples; SCZ (n = 60)***									
• GAF	0.017		0.010	1.693	0.096	0.000	0.010	0.002	0.998
• Age	0.027		0.017	1.551	0.127	0.009	0.017	0.556	0.581
• Sex (Male vs. Female)	−0.141		0.244	−0.577	0.567	−0.138	0.242	0.569	0.572
• BPRS	−0.019		0.008	−2.403	**0.020**	−0.002	0.008	−0.208	0.836
• Age	0.030		0.017	1.797	0.078	0.010	0.017	0.582	0.563
• Sex (Male vs. Female)	−0.232		0.244	−0.951	0.346	−0.130	0.246	−0.525	0.602
***Total samples; SCZ (n = 78)***									
• Antipsychotic dose	−0.000	0.000		−2.136	0.141	−0.000	0.000	−1.014	0.314
• Age	0.020	0.014		1.538	0.161	0.020	0.014	0.060	0.952
• Sex (Male vs. Female)	−0.284	0.197		−1.445	0.152	−0.096	0.195	−0.491	0.625

Abbreviations: s.e., standard error; SCZ, schizophrenia; GAF, Global Assessment Functioning; BPRS, Brief Psychiatric Rating Scale.

^a^β means standardized partial regression coefficient derived from generalized linear models.

^b^p value shown in bold is significant at < 0.05.

**Fig 2:**
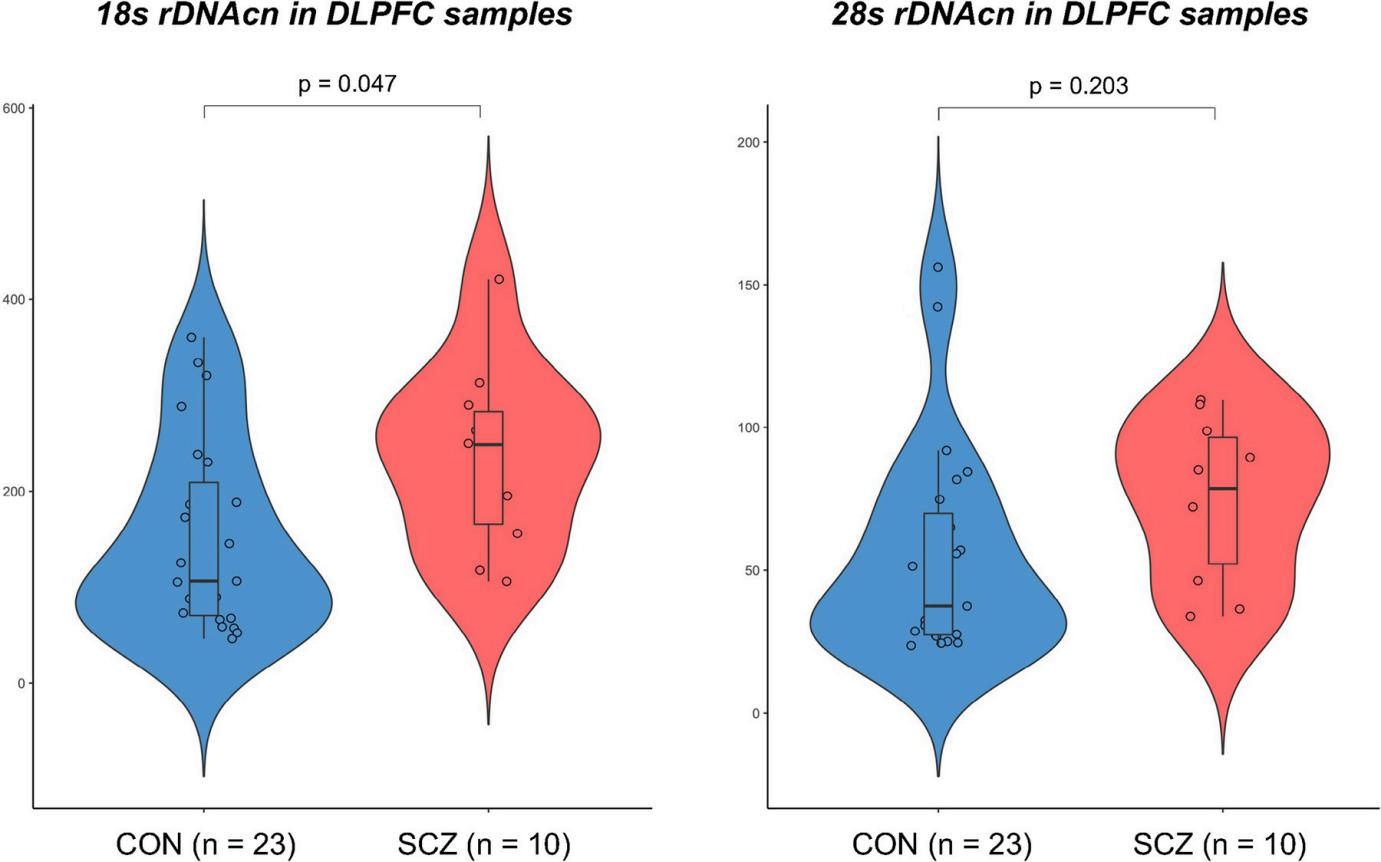
Violin and scatter dot plot including box and whiskers (with minimum to maximum) of 18S/28S ribosomal DNA copy number (rDNAcn) of dorsolateral prefrontal cortices (DLPFCs) in patients with schizophrenia (SCZ) and controls (CON). All p values were adjusted for age and sex as covariates.

The distribution of 18S/28S rDNAcn in the DLPFC samples was normalized (Kolmogorov–Smirnov test, p = 0.43). The results of multiple linear regression analyses of rDNAcn in DLPFC samples of patients with SCZ (n = 10) and healthy controls (n = 23), with age and sex as covariates, are shown in **S4 Table in S1 File**. These analyses showed a significant increase in 18S rDNAcn in the DLPFC of patients with SCZ compared to controls (SCZ: mean ± SD = 236.0 ± 91.2, min/max = 106.1/421.4; CON: 151.3 ± 97.4, 46.1/360.4; p  =  0.047), but not in 28S rDNAcn (SCZ: mean ± SD = 75.0 ± 26.8, min/max = 33.8/109.5; CON: 54.8 ± 36.1, 23.6/156.1; p  =  0.203) (**Fig 2**).

## Discussion

In this study, we successfully replicated the increased rDNAcn in the blood of patients with SCZ that was previously reported in Europeans using an ethnically different, East Asian, cohort. Furthermore, we provide the first evidence of increased rDNAcn in the brain of patients with SCZ.

To our knowledge, only one research group has previously reported the abundance of rDNA copies in patients with SCZ via their first report (SCZ n = 179) [7] and the second report with a larger sample size (SCZ n = 814) [8]. Given that their reports used only European samples, one of the main results of the present study is the confirmation of the increased rDNAcn in the genomes of patients with SCZ using an ethnically East Asian cohort, which strongly suggests that the increased rDNA content may be linked to a global and basic pathophysiology of SCZ. Indeed, recent large SCZ-genome-wide association studies focusing on European and East Asian populations have indicated that SCZ-risk genes and related pathophysiologies differ between Europeans and East Asians to some extent [12]. In such a situation, we believe that demonstrating the similarity in rDNA status of SCZ between two ethnicities will be very meaningful. In the subgroup analysis of blood samples, we found negative correlations between increased 18S rDNAcn and the severity of SCZ (nominal for higher GAF and statistically significant for lower BPRS). These results suggest that increased rDNA content can be a trait marker for SCZ but cannot be a state marker for its severity. Partially consistent with this interpretation, previous studies indicated that rDNA transcription in the dorsal raphe nucleus is only increased in residual SCZ, not in paranoid SCZ [5]. Furthermore, decreased rDNA transcription in the dorsal raphe nucleus in suicide victims (deemed as one of the most severe phenotypes of psychiatric diseases, including SCZ) regardless of psychiatric diagnosis is reported [13]. Meanwhile, we could not confirm the association between 28S rDNAcn and GAF/BPRS in patients with SCZ, suggesting that the subgroup analyses and results here were too preliminary to be treated as if on the same level as our main findings. While it appears that there were two subgroups within the patients with SCZ regarding blood rDNAcn distribution (in particular 28S rDNAcn as shown in **Fig 1**) with some patients showing aberrantly increased rDNAcn, we could not identify any clinical parameters to be statistically associated with this difference.

Another important finding of this study is the first demonstration of increased rDNAcn in the brains of patients with SCZ, since it is clear that the main diathesis of SCZ occurs in specific brain regions [14]. While previous studies showed that the transcriptional activity of rDNA in the dorsal raphe nucleus neurons was increased in patients with residual SCZ using the argyrophilic nucleolar organizer region silver staining method [4, 5], to date, no study has investigated rDNAcn in brain tissues of patients with SCZ. DLPFC is one of the brain regions which previous studies indicate is related to SCZ vulnerability [15]. There is consistent evidence that SCZ involves impaired functioning of the DLPFC, with deficits in working memory and DLPFC blood oxygen level-dependent responses that are related to the symptoms of thought disorders. It is also well documented that the expression of risk genes for SCZ is enriched in the prefrontal cortex compared to other brain areas. Structural and functional neuroimaging studies in populations with SCZ have revealed dysfunction of the DLPFC [16]. Given this evidence, aberrant rDNA content in the DLPFC of patients with SCZ, as shown in this study, may play an important role in DLPFC volume and functional abnormalities through dysfunctional neural plasticity caused by rDNA transcriptional activity. However, we should note that we could not confirm the association between increased 28S rDNAcn in the DLPFC of patients with SCZ same to 18S rDNAcn. Due to such difference observed between the 18S and 28S rDNAcn and the high variability among postmortem brain samples, brain analysis with larger sample sizes is needed to more precisely determine whether not only 18S rDNAcn but also 28S rDNAcn were increased in the DLPFC of patients with SCZ.

While the first preliminary demonstration of increased rDNAcn in the brains of patients with SCZ in this study might support the possibility that peripheral blood cells display the same genomic abnormalities than the affected tissue in the brain and can thus be utilized as a potential biomarker, no conclusions can be drawn from our preliminary findings in our brain analyses because of fundamental lack of sufficient sample size and data on the relationship between rDNAcn in bloods and that in brain tissues within same individuals.

The present study has several limitations. The first regards the use of post-mortem samples in our brain study. Although only individuals with certain clinical information prior to death were selected for inclusion, we were unable to exclude possible confounding effects of biological degradation of post-mortem samples in our results. In addition, we investigated clinical psychiatric scales and antipsychotic doses only in a part of the living patients whose blood samples were obtained, not in the postmortem patients whose DLPFC were obtained. Second, as stated in the previous study [7], qPCR works well on samples from living controls, but worse on living SCZ cases and even worse on postmortem samples due to the typical oxidative stress and resultant DNA breaks. This technique limitation of qPCR may have confounding effects on the following results in this study; 1) 18S and 28S rDNAcn of blood strongly correlated in controls, but moderately correlated in patients with SCZ, 2) in contrast to blood, the difference of rDNAcn in the postmortem brains between controls and patients with SCZ was marginal for 18S rDNAcn or at all insignificant for 28S rDNAcn, 3) rDNAcn in the postmortem brains appears to be lower than in the peripheral blood. Similarly, no reliable difference was found between control and SCZ rDNAcn in brain, and 28S rDNAcn in patients with SCZ did not have any association with GAF and BPRS scores, unlike 18S rDNAcn, which may be partially caused by the fact that DNA damage and hairpin formation is more prominent in 28S rDNA than 18S rDNA [7]. Therefore, future studies with hybridization-based techniques (such as dot hybridization) fitting better for rDNA quantification in SCZ patients and postmortem samples are needed. On the other hand, the ALB reference gene did not vary in patients with SCZ and controls in blood analysis as well as brain analysis (p > 0.05); this may provide reliability to some extent that our results directly reflect changes of rDNA contents in patients with SCZ. Third, average age of the healthy controls in our blood samples was statistically younger partially due to the inclusion of the students as our healthy control cohort. This might have a potential limitation because SCZ has late onset sometimes, and some of the students categorized as healthy controls at the timing of this study might develop SCZ in the future. Fourth, because of the available information from medical records, we cannot exclude other potential confounders, such as smoking status, which are known to affect rDNAcn [17]. Fifth, the sample sizes for blood and brain assessments in this study were too small to detect robust evidence for increased rDNA content in East Asian patients with SCZ. Sixth, the rDNAcn of other brain regions correlated with the pathophysiology of SCZ should be explored in the post-mortem brains of patients with SCZ. Finally, an important question whether increased rDNAcn in brain shown in this study translates to increased rRNA synthesis remains. Future research focusing on not only rDNA but rRNA in brains of same patients with SCZ is warranted.

## Conclusions

This is the first study to not only investigate and provide evidence demonstrating increased rDNAcn in the blood of patients with SCZ in East Asian populations, but we also provided the first evidence of increased rDNAcn in the DLPFCs of patients with SCZ. Our findings shed light on further research into rDNA towards an improved understanding of SCZ-related pathophysiology and brain-specific chromosomal abnormalities.

## Supporting information

S1 File (PDF)
