# Supplementary material for: Ribosomal DNA gene copies are increased in blood and brain of Japanese schizophrenia patients
Source: PLoS One. 2023 Jan 20;18(1):e0280694. doi: 10.1371/journal.pone.0280694 (PMC9858006; doi:10.1371/journal.pone.0280694)

S1 Table. Detailed description of GAF and BPRS

The Brief Psychiatric Rating Scale (BPRS)

BPRS has been widely used in schizophrenia research and validated to assess the psychopathology of schizophrenia, which a higher score means “more severe” [1, 2]. It consists of 18 symptom’s items graded on a 7-point Likert-type scale (0 = not assessed, 1 = not present, 2 = very mild, 3 = mild, 4 = moderate, 5 = moderately severe, 6 = severe, 7 = extremely severe). The 18 symptom’s items include somatic concern, anxiety, emotional withdrawal, conceptual disorganization, guilt feelings, tension, mannerisms and posturing, grandiosity, depressive mood, hostility, suspiciousness, hallucinatory behavior, motor retardation, uncooperativeness, unusual thought content, blunted affect, excitement, and disorientation.

The Global Assessment of Functioning (GAF)

GAF is a valid and reliable measure of various psychological disturbances and is used widely because of being able for evaluators to perform efficiently and quickly [3]. It quantifies the severity of psychiatry symptoms and levels of social functioning; the outcome is a single 100-point rating and represents lower points

as “more severe”. In addition, the reliability of GAF has been accepted among patients with schizophrenia [4].

References

1. Overall JE, Gorham DR. The Brief Psychiatric Rating Scale. Psychol Rep. 1962;10:799–812. https://doi.org/10.2466/pr0.1962.10.3.799.
2. Wang X, Yao S, Kirkpatrick B, Shi C, Yi J. Psychopathology and neuropsychological impairments in deficit and nondeficit schizophrenia of Chinese origin. Psychiatry Res. 2008;158:195-205. https://doi.org/10.1016/j.psychres.2006.09.007.
3. Jones SH, Thornicroft G, Coffey M, Dunn G. A brief mental health outcome scale-reliability and validity of the global assessment of functioning (GAF). Br J Psychiatry. 1995;166:654–9. https://doi.org/10.1192/bjp.166.5.654.
4. Startup M, Jackson MC, Bendix S. The concurrent validity of the Global Assessment of Functioning (GAF). Br J Clin Psychol. 2002;41(Pt 4):417-22. https://doi.org/10.1348/014466502760387533.

S2 Table. Primer sequences and polymerase chain reaction conditions

Target	Primers (5'-3')
18s rDNAcn	F: 5'-agcctgagaaacggctacca-3'<br>R: 5'-ggtcgggagtgggtaatttgc-3'<br>40 cycles of 95°C for 15 s, 58°C for 20 s, 72°C for 20 s
28s rDNAcn	F: 5'-gacctcagatcagaggtggcga-3'<br>R: 5'-ttcactcgccgttactgagggaat-3'<br>40 cycles of 95°C for 15 s, 58°C for 20 s, 72°C for 20 s
ALB	F: 5'-cggcggcgggcggcgcgggctgggcggaaatgctgcacagaatccttg-3'<br>R: 5'-gcccggcccgccgcgcccgtcccgccggaaaagcatggtcgcctgtt-3'<br>40 cycles of 95°C for 15 s, 58°C for 20 s, 72°C for 20 s

Abbreviations: rDNA, ribosomal DNA; ALB, albumin; min, minutes; s, seconds.

S3 Table. Results of regression analysis of rDNAcn in peripheral blood of patients with SCZ and controls

	18s rDNAcn				28s rDNAcn			
	β[a]	s.e.	t	p[b]	β[a]	s.e.	t	p[b]
Total samples (n = 179); SCZ (n = 81) and CON (n = 98)								
- Phenotype (SCZ vs. CON)	0.250	0.117	2.136	**0.034**	0.318	0.108	2.941	**0.004**
- Age	0.011	0.007	1.538	0.126	0.006	0.007	0.854	0.394
- Sex (Male vs. Female)	−0.041	0.117	−0.346	0.730	−0.165	0.108	−1.522	0.130

Abbreviations: s.e., standard error; SCZ, schizophrenia; CON, control.

[a]β means standardized partial regression coefficient derived from generalized linear models.

[b]p value shown in bold is significant at < 0.05.

S4 Table. Results of regression analysis of rDNAcn in DLPFC of patients with SCZ and controls

	18s rDNAcn				28s rDNAcn			
	β[a]	s.e.	t	p[b]	β[a]	s.e.	t	p[b]
Total samples (n = 33); SCZ (n = 10) and CON (n = 23)								
- Phenotype (SCZ vs. CON)	0.346	0.168	2.078	**0.047**	0.224	0.203	1.303	0.203
- Age	−0.196	0.177	−1.255	0.220	−0.203	0.183	−1.261	0.217
- Sex (Male vs. Female)	0.353	0.156	2.382	**0.024**	0.382	0.162	2.500	**0.018**

Abbreviations: DLPFC, dorsolateral prefrontal cortex; s.e., standard error; SCZ, schizophrenia; CON, control.

[a]β means standardized partial regression coefficient derived from multiple linear regression.

[b]p value shown in bold is significant at < 0.05.

S1 Fig. Association between 18S and 28S rDNAcn in peripheral blood of patients with SCZ and controls

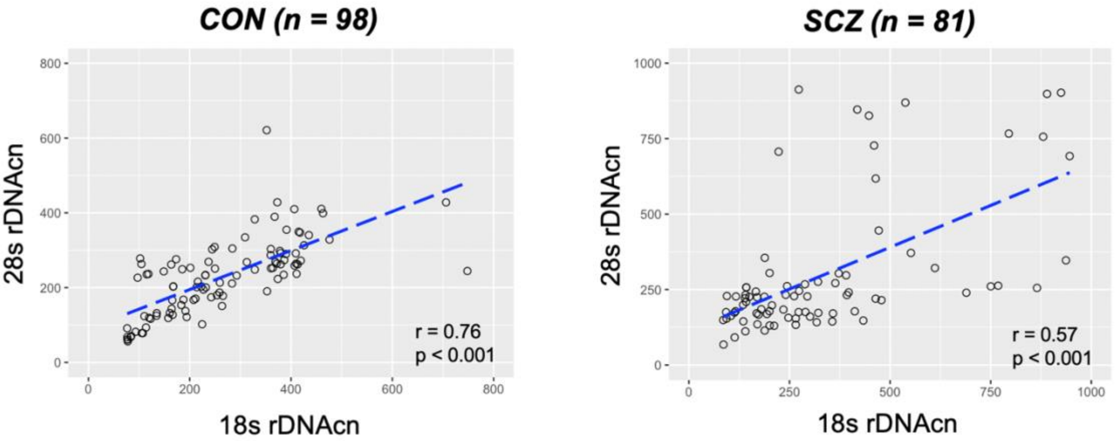

S2 Fig. Association between 18S and 28S rDNAcn in DLPFC of patients with SCZ and controls

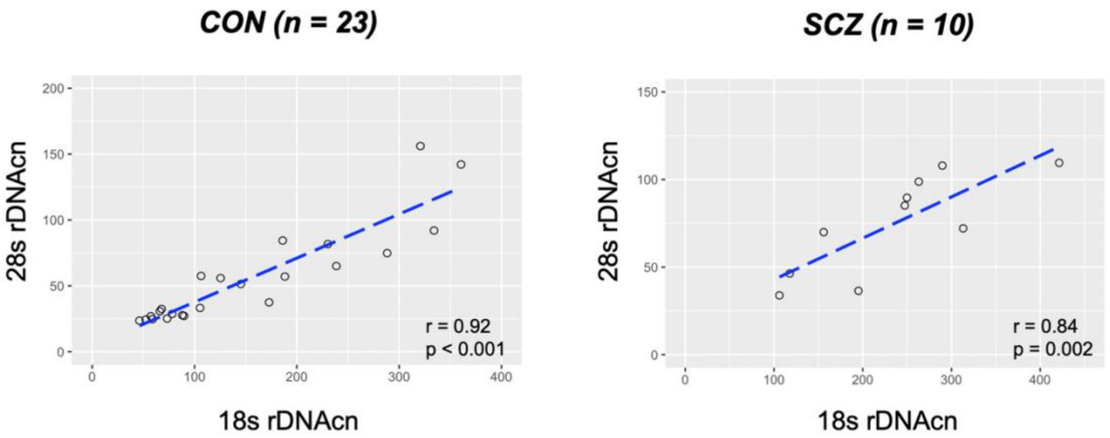

Supplement: S1 File — (PDF) [file pone.0280694.s001.pdf]
